# AIDS knowledge and attitudes in a Turkish population: an epidemiological study

**DOI:** 10.1186/1471-2458-5-95

**Published:** 2005-09-13

**Authors:** Unal Ayranci

**Affiliations:** 1 Medico-Social Center, Osmangazi University, 26480 Meselik-Eskisehir, Turkey

## Abstract

**Background:**

The aim of this study was to investigate and present some pertinent comments concerning Acquired Immunodeficiency Syndrome (AIDS) knowledge, attitudes and misconceptions among the general population in a city of west Turkey. This study was deemed important and relevant due to the increasing importance of AIDS in Turkey and the other countries.

**Methods:**

Using a multistage area sampling method, a random sample of individuals aged 11–83 years, living in 65 different quarters in the city of Eskisehir, Turkey during September, October and November 2004 were interviewed.

**Results:**

In all, 1048 respondents completed the survey. In most items, respondents displayed a fairly good to excellent degree of knowledge about HIV/AIDS. Individuals with higher degrees of education indicated more correct responses in all items relating to knowledge of HIV/AIDS.

In general, the respondents' attitudes towards AIDS and people with AIDS were found to be tolerant and positive, with one answer choice showing that the majority of the respondents agreed with the statement that those with HIV/AIDS must be supported, treated and helped (90.7%). Moreover, the proportions of the respondents' misconceptions were found to be significantly low for all the items. However, nearly one fourth of the respondents agreed with the misconceptions 'AIDS is a punishment by God' and 'One is not infected with HIV/AIDS if engaged in sport and well nourished'.

**Conclusion:**

In general HIV/AIDS related knowledge was high and people showed positive attitudes. However, people continue to hold misconceptions about AIDS and these need to be addressed by health education programs targeting those at higher risk.

## Background

The Acquired Immunodeficiency Syndrome (AIDS), one of the most complex health problems of the 21 st century, is in its third decade and has become a pandemic disease that threatens the world population. Moreover, with no treatment or cure in sight, the disease continues to spread at an alarming rate [1-3]. Recent epidemiological data indicates that an estimated 34–46 million individuals are living with Human Immunodeficiency Virus (HIV)/AIDS [2-4]. Over 30 million people have already died from AIDS, with the year 2003 alone seeing 3 million [4]. Four million children have been infected since the virus first appeared. Over 90% of these individuals are concentrated in the developing countries, mostly in countries least able to afford to care for infected people. Over 50% of the newly infected adults are in the age bracket 15 to 24 years old and more than 40% are women [2,4-6].

In Turkey, the importance of AIDS started with the diagnosis of two patients in the year 1985, and still continues to be on the agenda up to the present day. The number of AIDS cases increases every year: 34 new cases in 1990, 91 new cases in 1995, and 119 new cases in 1999. According to the June 2004 statistics of the Ministry of Health (MoH) the cumulative number of HIV positive patients is 1802, 76% of which are sexually active and social individuals aged between 15–49 years. Approximately 800 of these were AIDS patients [7]. These numbers, however, are only the official numbers of the MoH. As has been previously established, the number of patients with infectious diseases increases geometrically. Thus, based on the original two cases of 1985, the actual number of the patients in 2004 has been calculated at about 10,000. According to the information supplied by the MoH, AIDS in this country is considered to be in its beginning phase, compared with the calculation above. However, this figure does not reflect the real numbers afflicted due to the inadequacy of the registration system and the fact that people with sexually transmitted diseases do not generally attend health centers [8].

This article presents data from a study of the general population of Eskisehir, a city in western Turkey. Several studies, conducted among selected target populations, have evaluated HIV/AIDS knowledge and the attitudes of certain groups such as university students [9], health non-commissioned officer candidates [10], soldiers [11], street children/youths [12], and nurses [13] within the Turkish society. In Turkey, however, we are unaware of any earlier studies of HIV/AIDS-related knowledge, attitudes, and misconceptions or practices conducted among the general population of Turkey. This was the particular aim of this study. Therefore, the present study sought to address Turkish society's risk behavior, knowledge, attitudes and misconceptions about HIV/AIDS, to assess needs for sex education and to discover sources of information about AIDS for the people in a city of west Turkey.

## Methods

### Setting

Eskisehir is a semi rural province situated in the western part of Turkey, with a population of about 460,000. The socio-economical level of the city is average compared to other cities of the country. There are significant disparities in the socio-economic characteristics between the quarters of the city. It includes two universities, and also has a cosmopolitan structure.

### The questionnaire

The questionnaire used in this survey, based on the WHO AIDS programme knowledge, attitudes, beliefs and practices (KABP) survey in 1988 [14] as well as literature [3,15-20], was modified to suit the Turkish culture and norms, in a way that covered all the profession groups in the general public living in the city, such as students, tradesmen, workers, housewives, lorry drivers etc. The questionnaire, consisting of 56 questions in Turkish, was divided into three broad sections: sociodemographic characteristics, knowledge concerning AIDS, and sources of information about AIDS. The sources of information included modes of transmission, attitudes towards AIDS and people with AIDS, and misconceptions or beliefs about AIDS and AIDS patients. Some questions on the questionnaire, such as those concerning the source of information of HIV/AIDS, were open-ended and covered sociodemographic characteristics, knowledge of the disease including the mode of transmission and populations at high risk, attitudes towards HIV-positive patients, and the source of knowledge and beliefs towards HIV/AIDS. The questionnaire was then pre-tested on a sample of 62 participants from different subpopulations of the city. Alpha coefficients for reliability and internal consistency of the questions were found to be 0.891, 0.734, and 0.603 for knowledge, attitudes, beliefs or misconceptions about HIV/AIDS, respectively. The completed questionnaires were checked for consistency and completeness. Questions were answered using the options "Agree/True", "Disagree/False", and "I don't know/I have no idea". Responses to all the items were converted to a percentage indicating the proportion of correct responses.

### Sampling

1048 people were interviewed face-to-face between September and November 2004 for a study on the KABP relating to HIV/AIDS of the population in a city of western Turkey.

Due to the questionnaire being rather long, the survey being conducted on a general population and there being a possibility that some people in the city are illiterate, trained interviewers (4 females and one male) helped to explain any questions that the respondents found incomprehensible. 274 houses and 108 workplaces situated in the 65 quarters of the city, each having approximately equal populations, were determined using a stratified random sample method. During the study period, a total of 1,621 people were working or living in these places, with 479 people living in houses, and 1,142 people working in workplaces. The study was conducted at the participants' work place or home. Our objective was to contact the whole population of subjects in the aforementioned places. Criteria for inclusion in the study was having the ability to complete the questionnaire and completion of education to at least primary school level, working on the presumption that this would ensure that all participants had a basic knowledge level of sexuality, a basic level of maturity with regard to answering sexually related questions, or the ability to communicate with one another. Those who came to visit the city from other cities and those with hearing impairments were excluded from the study. In addition, children and those not willing to participate were also excluded. The sample was representative. It did not differ from the general population in terms of age or sex.

### Procedures

All subjects (1048/1621, 64.7%) were told that participation in the investigation was strictly voluntary and were told that the data collected would not be used for anything except the research aim. Those who agreed to participate were given the questionnaire to complete. The duration for completing the questionnaire was between 20–25 minutes per subject. The principal investigator met weekly with the data collectors to ensure the quality of data collected.

### Legal ethical consent

Ethical permission for the study was obtained prior to collect data, by contacting and receiving approval from the appropriate management authority, the health directorship of the city involved. Participants were assured of the confidentiality of their responses and provided informed verbal consent.

### Statistical analyses

The statistical package for social sciences (SPSS) version 10.0 (Chicago, IL, USA) was used to enter and analyze the data on a personal computer. Obtained data were evaluated by frequency and percentages ratios, Chi-square (x^2^) and *t *tests. The measure for statistical significance was established a priori as *P *< 0.05.

## Results

### Sample characteristics

The mean (± SD) age of the respondents (n = 1048) was 29.9 ± 10.6, 95% CI (Confidence Interval) of 29.3–30.6, ranging from 11 to 83 years. It was significantly lower in women than in men (28.6 ± 10.9, 95% CI of 27.6–29.6 and 31.1 ± 10.3, 95% CI of 30.2–31.9, respectively), (t = 3.7, d.f. = 1046, p = 0.000, 95% CI of 1.13–3.71). Age ranged from 11 to 83 in men and from 12 to 76 in women. More respondents (56.7%) were male and single (48.1%) or married (46.9%), had attained the level of secondary education or above (86.2%). Most participants (64.8%) were working in a job and believed in God (87.9%). Household income levels were average or higher (71.6%). The characteristics of the participants are presented in Table 1.

**Table 1:** The respondents' characteristics (n = 1048)

	**Number**	**Percentage**
Gender		
Male	594	56.7
Female	454	43.3
Age		
≤24	396	37.8
25–34	367	35.0
35–44	184	17.6
≥45	101	9.6
Employment status		
Employed	679	64.8
Housewife	155	14.8
Student	83	7.9
Unemployment	59	5.6
Retired	72	6.9
Number of those living at home		
Between 1–3	345	32.9
Between 4–5	552	52.7
≥6	151	14.4
Marital status		
Single	504	48.1
Married	492	46.9
Widowed/divorced/seperated	52	5.0
Presence of religious belief		
Yes	921	87.9
No	98	9.4
Unsure	29	2.8
Educational levels		
Illiterate	20	1.9
Primary	125	11.9
Secondary	119	11.4
High school	280	26.7
Higher education	504	48.1
Family's total income level		
Low	298	28.4
Average	521	49.7
High	229	21.9

Sixty two percent of the sample believed they were not at any risk of contracting HIV (Unshown data).

### Respondents' knowledge levels

The analysis of data indicated that in most items respondents had a fairly good to excellent knowledge about HIV/AIDS. The percentages of 'true' responses for all the knowledge items were higher than 'false' and 'don't know' responses, with the exception of the response for item 7. The vast majority had correct knowledge about items 2 (92.0%), 25 (94.3%), 28 (93.6%), 29 (91.5%), and 30 (90.6%). However, over 30% of the respondents thought that AIDS is a hereditary disease (37.6%); that AIDS is not generally seen in developing or underdeveloped countries (38.5%); that a person infected with HIV usually shows some symptom of the disease (39.2%); and that urine, X-ray, total blood count and biochemistry analyses are the tests used to check for the HIV virus in the blood (30.0%). Further misconceptions held were that HIV/AIDS can be contracted through sharing public toilets and swimming pools with an infected person (33.4%); using an infected person's belongings such as clothes, comb, underwear and towels (33.7%); sharing the food utensils of an infected person (37.7%); and exposure to an infected person who coughs or spits (34.9%), or the urine of an infected person (31.2%).

There was an important evidence of sex differences in responses regarding knowledge about HIV/AIDS for 13 items out of the 34. In statements relating to knowledge on items 1., 2., 3., 4., 6., 9., 11., 21., 22., 23., 29., 32., and 33, males gave significantly more correct responses than did female respondents.

Age groups were found to be significantly associated with AIDS related knowledge for 16 of the items. Younger respondents (24 years) responded better to items 6., 16., 17., and 31., whereas older respondents (45 years) responded better to items 5., 7., 8., 26., and 35. On the other hand, those in age group 25–34 years old had more correct responses for items 4., 9., 10., 12., and 20., and those in age group 35–44 years old had significantly more correct responses for items 19., and 27. The detailed data are presented in Table 2.

**Table 2:** The respondents' knowledge on HIV/AIDS

**Knowledge items (Alpha 0.891 for the below 34 items)**	**Yes n(%)**	**No n(%)**	**Don't know n(%)**
**General knowledge:**			
1 A virus causes AIDS	794(75.8)√	87(8.3)	167(15.9)
2 AIDS is a contagious disease	964(92.0)√	46(4.4)	38(3.6)
3 AIDS is a hereditary disease	394(37.6)	496(47.3)√	158(15.1)
4 There is an active treatment for AIDS	305(29.1)	545(52.0)√	198(18.9)
5 AIDS is mostly seen in the developing or underdeveloped countries, mostly in countries least able to afford to care for infected people	511(48.8)√	403(38.5)	134(12.8)
6 AIDS is not a serious disease. It is a simple disease like the common cold	85(8.1)	904(86.3)√	59(5.6)
7 A person infected with HIV does not usually show any symptoms of the disease	340(32.4)√	411(39.2)	297(28.3)
8 Resistance to other diseases in an individual with AIDS is rather low	727(69.4)√	138(13.2)	183(17.5)
9 There is a vaccine for AIDS	152(14.5)	737(70.3)√	159(15.2)
10 We can distinguish AIDS patients from others by their appearance	242(23.1)	561(53.5)√	245(23.4)
11 The ELISA test is used to check for the HIV virus in the blood	700(66.8)√	92(8.8)	256(24.4)
12 Urine, X-ray, total blood count and biochemistry analyses are used to check for the HIV virus in the blood	314(30.0)	433(41.3)√	301(28.7)
***HIV/AIDS can be contacted through:***			
13 Sharing public toilets and swimming pools with an infected person	350(33.4)	560(53.4)√	138(13.2)
14 Using an infected person's belongings such as clothes, comb, underwear and towel	353(33.7)	568(54.2)√	127(12.1)
15 Sharing a razor blade with an infected person	780(74.4) √	164(15.6)	104(9.9)
16 Touching an infected person, such as hugging, holding and shaking hands	243(23.2)	718(68.5)√	87(8.3)
17 Sharing the food utensils of an infected person	395(37.7)	510(48.7)√	143(13.6)
18 Exposure to an infected person who coughs or spits	366(34.9)	531(50.7)√	151(14.4)
19 Having a tattoo done with the same devices after an infected person	798(76.1)√	122(11.6)	128(12.2)
20 The bite of a mosquito	312(29.8)	468(44.7)√	268(25.6)
21 Sharing injection needles or the surgical operation devices of an infected person	933(89.0)√	50(4.8)	65(6.2)
22 Having a tooth extracted with the same devices after an infected person	896(85.5)√	53(5.1)	99(9.4)
23 An infected pregnant woman's infecting her unborn baby	816(77.9)√	82(7.8)	150(14.3)
24 Donating to another person the organs and tissue of an infected person	819(78.1)√	84(8.0)	145(13.8)
25 Having vaginal sex with an infected person	988(94.3)√	26(2.5)	34(3.2)
26 Having oral sex with an infected person	741(70.7)√	148(14.1)	159(15.2)
27 Having anal sex with an infected person	821(78.3)√	76(7.3)	151(14.4)
28 Receiving blood from an infected person	981(93.6)√	28(2.7)	39(3.7)
29 The vaginal liquid of an infected person	959(91.5)√	26(2.5)	63(6.0)
30 The sperm of an infected person	950(90.6)√	45(4.3)	53(5.1)
31 The urine of an infected person	327(31.2)	505(48.2)√	216(20.6)
32 The tears of an infected person	171(16.3)	659(62.9)√	218(20.8)
33 The mucus or nasal fluid of an infected person	167(15.9)	652(62.2)√	229(21.9)
34 The breast milk of an infected person	657(62.7)√	191(18.2)	200(19.1)

√ True responses

Table 3 shows the respondents' AIDS knowledge levels according to their educational status. It revealed that the respondents' AIDS knowledge levels showed statistical significances according to their educational status for all but 8 knowledge items (5., 7., 20., 21., 26., 27., 29. and 30.). The knowledge levels of those who had attained a higher education level, such as university, were higher than those having lower educational levels, save for 6 items out of 34 (7., 26., 27., 29., 30., and 34.).

**Table 3:** AIDS knowledge levels by educational status of the respondents

General knowledge items on HIV/AIDS and the proportions of those who answered correctly	**Illiterate n(%) 20(1.9)**	**Primary n(%) 125(11.9)**	**Secondary n(%) 119(11.4)**	**High school n(%) 280(26.7)**	**Higher school n(%) 504(48.1)**	**Those answering correctly n(%) 1048(100.0)**
1 A virus causes AIDS‡	15(75.0)	72(57.6)	65(54.6)	203(72.5)	439(87.1)	794(75.8)
2 AIDS is a contagious disease‡	18(90.0)	108(86.4)	106(89.1)	249(88.9)	483(95.8)	964(92.0)
3 AIDS is a hereditary disease‡	6(30.0)	34(27.2)	37(31.1)	125(44.6)	294(58.3)	496(47.3)
4 There is an active treatment for AIDS‡	8(40.0)	50(40.0)	44(37.0)	153(54.6)	290(57.5)	545(52.0)
5 AIDS is mostly seen in developing or underdeveloped countries, mostly in countries least able to afford to care for infected people*f*	9(45.0)	62(49.6)	50(42.0)	138(49.3)	252(50.0)	511(48.8)
6 AIDS is not a serious disease. It is a simple disease like the common cold‡	13(65.0)	93(74.4)	83(69.7)	237(84.6)	478(94.8)	904(86.3)
7 A person infected with HIV does not usually show any symptoms of the disease*f*	10(50.0)	40(32.0)	29(24.4)	86(30.7)	175(34.7)	340(32.4)
8 Resistance to the other diseases in an individual with AIDS is rather low†	12(60.0)	87(69.6)	68(57.1)	198(70.7)	362(71.8)	727(69.4)
9 There is a vaccine for AIDS‡	8(40.0)	56(44.8)	62(52.1)	195(69.6)	416(82.5)	737(70.3)
10 We can distinguish AIDS patients from others by their appearance‡	7(35.0)	39(31.2)	51(42.9)	144(51.4)	320(63.5)	561(53.5)
11 The ELISA test is used to check for the HIV virus in the blood‡	10(50.0)	58(46.4)	59(49.6)	165(58.9)	408(81.0)	700(66.8)
12 Urine, X-ray, total blood count and biochemistry analyses are used to check for the HIV virus in the blood‡	3(15.0)	33(26.4)	38(31.9)	84(30.0)	275(54.6)	433(41.3)
*HIV/AIDS can be contacted through:*						
13 Sharing public toilets and swimming pools with an infected person‡	8(40.0)	43(34.4)	51(42.9)	145(51.8)	313(62.1)	560(53.4)
14 Using an infected person's belongings such as clothes, comb, underwear and towel‡	5(25.0)	45(36.0)	51(42.9)	139(49.6)	328(65.1)	568(54.2)
15 Sharing a razor blade with an infected person‡	13(65.0)	85(68.0)	72(60.5)	203(72.5)	407(80.8)	780(74.4)
16 Touching an infected person, such as hugging, holding and shaking hands‡	9(45.0)	54(43.2)	60(50.4)	182(65.0)	413(81.9)	718(68.5)
17 Sharing the food utensils of an infected person‡	6(30.0)	33(26.4)	39(32.8)	123(43.9)	309(61.3)	510(48.7)
18 Exposure to an infected person who coughs or spits‡	7(35.0)	39(31.2)	45(37.8)	121(43.2)	319(63.3)	531(50.7)
19 Having a tattoo done with the same devices after an infected person‡	13(65.0)	91(72.8)	76(63.9)	206(73.6)	412(81.7)	798(76.1)
20 The bite of a mosquito*f*	7(35.0)	49(39.2)	52(43.7)	126(45.0)	234(46.4)	468(44.7)
21 Sharing the injection needles or surgical operation devices of an infected person*f*	15(75.0)	110(88.0)	95(79.8)	243(86.8)	470(93.3)	933(89.0)
22 Having a tooth extracted with the same devices after an infected person†	15(75.0)	107(85.6)	95(79.8)	228(81.4)	451(89.5)	896(85.5)
23 An infected pregnant woman's infecting her unborn baby†	15(75.0)	91(72.8)	84(70.6)	207(73.9)	419(83.1)	816(77.9)
24 Donating the organs and tissue of an infected person to another person†	15(75.0)	91(72.8)	83(69.7)	211(75.4)	419(83.1)	819(78.1)
25 Having vaginal sex with an infected person†	18(90.0)	117(93.6)	105(88.2)	261(93.2)	487(96.6)	988(94.3)
26 Having oral sex with an infected person†	13(65.0)	100(80.0)	85(71.4)	211(75.4)	332(65.9)	741(70.7)
27 Having anal sex with an infected person*f*	13(65.0)	102(81.6)	91(76.5)	220(78.6)	395(78.4)	821(78.3)
28 Receiving blood from an infected person‡	18(90.0)	114(91.2)	102(85.7)	260(92.9)	487(96.6)	981(93.6)
29 The vaginal liquid of an infected person*f*	16(80.0)	116(92.8)	104(87.4)	259(92.5)	464(92.1)	959(91.5)
30 The sperm of an infected person*f*	18(90.0)	111(88.8)	104(87.4)	263(93.9)	454(90.1)	950(90.6)
31 The urine of an infected person‡	9(45.0)	42(33.6)	46(38.7)	131(46.8)	277(55.0)	505(48.2)
32 The tears of an infected person‡	10(50.0)	62(49.6)	62(52.1)	167(59.6)	358(71.0)	659(62.9)
33 The mucus or nasal fluid of an infected person‡	10(50.0)	56(44.8)	61(51.3)	163(58.2)	362(71.8)	652(62.2)
34 The breast milk of an infected person†	8(40.0)	56(44.8)	46(38.7)	112(40.0)	150(29.8)	657(62.7)

p < 0.001‡, p > 0.05*f*, p < 0.05†

### Respondents' attitudes

In general, the respondents' attitudes towards AIDS and people with AIDS were found to be significantly tolerant and positive, with the exception of items 7 and 8. The majority of the respondents positively agreed with statement 5 (70.5%) and statement 9 (90.7%). On the other hand, a large number of the respondents agreed with the stigmas 1., 2., 3., 4., 6., 7., 8 and 10 with proportions of between 30% and 50%. These results are shown in Table 4.

**Table 4:** The respondents' attitudes towards HIV/AIDS

**Attitudes to persons with HIV/AIDS (Alpha 0.734 for the below 10 items)**	Agree n(%)	Disagree n(%)	Neither agree nor disagree n(%)
1 Students with AIDS should go to special schools for those with AIDS	389(37.1)	504(48.1)√	155(14.8)
2 If there is a student with AIDS in a school, I would delete the record of my child from that school	346(33.0)	554(52.9)√	148(14.1)
3 I would not sit in the same armchair or desk with a person with AIDS	345(32.9)	588(56.1)√	115(11.0)
4 I would not kiss someone with AIDS	400(38.2)	553(52.8)√	95(9.1)
5 They should be locked up or isolated in a special center	187(17.8)	739(70.5)√	122(11.6)
6 I would have personal contact with someone with AIDS as an ordinary person	601(57.3)√	333(31.8)	114(10.9)
7 I would share public toilets and swimming pools with someone with AIDS	412(39.3)√	506(48.3)	130(12.4)
8 I would wash my clothes with those of an individual with AIDS	437(41.7)√	489(46.7)	122(11.6)
9 They must be supported, treated and helped	951(90.7)√	50(4.8)	47(4.5)
10 Everybody must know about those with AIDS by means of national media	332(31.7)	579(55.2)√	137(13.1)

√ Positive attitudes

Women were more positive in their attitudes towards HIV/AIDS or AIDS victims when answering all the items compared to men, and those attitudes were statistically significant for items 1., 3., 5., 8., and 10.

There were significant differences for items 2., 3., 4., and 7 between different age groups with regard to their attitudes towards AIDS and AIDS victims. Those in the age group 45 years old and above had significantly more positive attitudes to items 2., and 7., whereas those aged 24 years old and under had significantly more positive attitudes to items 3., and 4.

Upon comparison of people with different educational levels, it was found that there were significant differences between individuals with different levels of education for 8 items out of 10. Those with higher education were significantly more positive in their attitudes to items 1., 2., 3., 9., and 10., compared to less educated respondents, whereas illiterate individuals had significantly more positive attitudes to items 4., 5., and 7 when compared to higher educated respondents.

### Respondents' misconceptions

In general, the proportions of the respondents' misconceptions were found to be significantly low for all the items. The majority of the respondents disagreed with all the statements. However, 23.2% and 24.2% agreed with misconceptions 2., and 6., respectively. The results are shown in Table 5.

**Table 5:** The respondents' misconceptions towards HIV/AIDS

**Misconceptions to persons with HIV/AIDS (Alpha 0.603 for the below 6 items)**	Agree n(%)	Disagree n(%)	Neither agree nor disagree n(%)
1 If you are passionately in love with someone, you become immune to AIDS	73(7.3)	930(88.7)	45(4.3)
2 AIDS is a punishment from God	243(23.2)	707(67.5)	98(9.4)
3 AIDS does not influence the Turkish	46(4.4)	982(93.7)	20(1.9)
4 I will not be infected with AIDS come what may	100(9.5)	884(84.4)	64(6.1)
5 Married couples do not contract AIDS even if they have sex with others	43(4.1)	957(91.3)	48(4.6)
6 You cannot be infected with HIV/AIDS if you are engaged in sport and are well nourished	254(24.2)	620(59.2)	174(16.6)

Women more disagreed with the vast majority of the misconceptions, with the exception of item 4., where men more disagreed. There were significant differences between men and women to the misconception items 4., and 5..

Significant differences between different age groups were seen for only 2 of the items (2., and 6.) with regard to their misconceptions towards AIDS and AIDS victims. Those in the age group 24 years old and under disagreed more significantly with misconception item 2., whereas those in the age group 35–44 disagreed more significantly towards misconception item 6. However, no difference was observed between the other items (1., 3., 4., and 5.) and different age groups.

The proportions of those who neither agreed nor disagreed were higher for those agreeing with all items; however, there were no significant differences between religious belief and misconceptions, except for item 6.

Although the proportions of those who agreed with all the items were higher in men than in women, there were significant differences between religious beliefs and sex differences, with the exception of items 4., and 5.

There were no significant differences between different age groups and those who agreed with misconceptions towards AIDS and AIDS victims, barring item 2 where the proportion of those agreeing with item 2 was higher in those aged 45 years and over when compared to the other age groups.

While comparing people with different educational levels, it was found that there were significant differences between individuals with different levels of education in all of the misconception items.

Those with higher education disagreed significantly more with all the misconceptions, compared to less educated respondents.

### Sources of HIV/AIDS information

The majority of the respondents indicated that their level of information about HIV/AIDS was average (49.3%). Most respondents reported that mass media (television, 68.9%; newspapers, 47.6%; magazines, 23.6%) was the major sources of their information about HIV/AIDS, followed by school and friends (18.8% and 18.7%, respectively). However, most indicated that they desired to learn more (86.6%). These results are shown in Table 6

**Table 6:** The respondents' source of information and their informational needs (n = 1048)

	Number*	**Percentage**
**Level of information about HIV/AIDS**		
Average	517	49.3
Bad	446	42.6
Good	85	8.1
**Source of information**		
Television	722	68.9
Newspaper	499	47.6
Magazine	247	23.6
School	197	18.8
Friend	196	18.7
Book	154	14.7
Radio	141	13.4
Doctor	119	11.3
Teacher	82	7.8
No	70	6.7
Family	49	4.7
Nurse	47	4.5
Internet	19	1.8
Poster	16	1.5
Seminar	14	1.3
AIDS Association	8	0.7
Workplace	3	0.2
**Desire to learn more**		
Yes	908	86.6
No	140	13.4

*The total exceeds the sample size since each respondent could choose several response categories

## Discussion

This paper reports data from a population-based study on AIDS knowledge, attitudes and misconceptions among the general population in Eskisehir, Turkey. The findings indicated that people in Turkey reported good knowledge about AIDS. However, nearly 40% and 30% of the respondents believed that AIDS is a hereditary disease and that there is an active treatment for AIDS, respectively. They also indicated that they believed that AIDS is not generally seen in the developing or underdeveloped countries, countries that would be least able to afford to care for infected people (38.5%), that people infected with HIV usually show some symptom of the disease (39.2%), and that 23.1% of them believed that AIDS patients differ from the normal population in their appearance (23.1%). Furthermore, respondents demonstrated a limited knowledge of how HIV/AIDS cannot be transmitted whereas a rather high rate said that AIDS could be contracted through items 13 (33.4%), 14 (33.7%), 17 (37.7%), 18 (34.9%), 20 (29.8%), and 31 (31.2%). It appears that a number of respondents in the city do not know about the risk of transmission of AIDS or HIV infection from different sources. These findings are consistent with other results in both our country and other countries [9,15,17,21].

The findings suggested that gender was not associated with the correct answering of questions on AIDS-related knowledge, excluding those of 13 items (1., 2., 3., 4., 6., 9., 11., 21., 22., 23., 29., 32., and 33.) where males preformed better. In contrast, as expected, those who had higher levels of education and younger respondents (those aged 34 and under) generally had more correct answers on questions relating to knowledge about AIDS. A study from the US among the general population also showed significant differences in AIDS-related knowledge with less-educated and older respondents being less likely to respond correctly to general AIDS knowledge questions [22]. Another reason for the higher proportion of true answers in men than women may be that men feel freer than women to talk about matters relating to sex and HIV/AIDS [23]. Furthermore, there were significant differences between those with different levels of education and knowledge about AIDS. In all items individuals with higher education levels, especially those with a university or college education, had more correct responses for 28 of the items, barring only 6 items (7., 26., 27., 29., 30., and 34.).

The most interesting finding from this survey was the fact that people in the city showed a more positive attitude towards AIDS and those with AIDS than expected, with the exception of a few items (7., and 8.). For example only a small number of the respondents disagreed with the attitude that people with AIDS must be supported, treated and helped (4.8%) or agreed with the attitude that people with AIDS should be locked up or isolated in a special center (17.8%). Furthermore, when taking into consideration that the majority of the respondents' educational levels were high school and above (74.8%) and that their levels of information about HIV/AIDS were average or above (57.4%) our study, in line with the study of Maswanya et al (2000) [24], may conclude that people with good knowledge or good education about AIDS do become more tolerant of people with AIDS.

On the other hand, there was a substantial negative attitude towards HIV and AIDS positive patients. A proportion of about 30% and 50% of the respondents, excluding item 5 where the proportion of negative attitude was only 17.8%, expressed negative attitudes. These findings are consistent with the findings of some studies conducted in Iran and Indian [3,23]. This can be explained by the similar sociocultural design of Turkish, Iranian and Indian attitudes towards HIV/AIDS, especially in the light of religious factors, and may also be explained by the respondents having confused opinions towards HIV virus and people with AIDS.

In our study, 17.8% of the respondents agreed with the statement suggesting that people with AIDS should be locked up or isolated in a special center. This result was in line with the study by Moatti et al (1998) [25], where the proportion of this attitude was 21.9%. One explanation for this attitude may be that some people in the community do not know much about AIDS or people with AIDS.

In this study, women were more positive in their attitudes towards HIV/AIDS or AIDS victims in all the items when compared to men. This finding is compatible with the study by Lester (1989) [26]. This may be explained by women being more sensitive due to naturally possessing the mothering instinct.

This survey demonstrated that in general only a small number of the respondents harbored misconceptions. For instance, only 23.2% of people in a Muslim country such as Turkey agreed with the statement that AIDS is a punishment from God. In parallel, this proportion was 14.2% in Montazeri (2004)'s study [20] conducted in Iran and 30.2% in the study conducted out by Tebourski and Alaya (2004) [15] in Tunisia. This means that these people believe that even religious factors could not prevent a person from HIV infection or simply that they do not believe that holding religious beliefs can be preventive. However, Nwokoji and Ajuwon (2004) [17] indicated that such misconceptions might encourage some individuals to take risks by creating the false impression that they will be cured if they become infected with AIDS [17]. It seems there is a need for further investigation into the role of religion in AIDS prevention, particularly in countries such as Turkey where religion plays an important role in people's everyday life.

In the current study, those having attained a higher education level disagreed significantly more with all the misconceptions, compared to less educated respondents. This is compatible with the study of Eshetu et al (2004) [27]. This indicates that raising educational levels is a key tool in fighting the epidemic.

In this study, the most important AIDS related knowledge source for the majority of the respondents was the mass media such as television (68.9%) and newspapers (47.6%), followed by magazines (23.6%). It appears that the mass media, especially television, has an important role in raising AIDS awareness within the Turkish community. In contrast, institutions such as school (18.8%) and the AIDS Association (0.7%) where better information about AIDS can be found, or from specialist persons such as doctors (8.1%), teachers (7.8%) and nurses (4.5%) had less importance. These should be more involved in AIDS education. In addition, only 4.5% said that their family informed them about the disease. The sources of knowledge of HIV/AIDS for the general population reported here are similar to those previously reported [9,11,16,28]. To explain these findings, we may say that the importance of the mass media is obvious as a means of maintaining information about AIDS-related problems, and also that very little communication regarding HIV/AIDS occurred between themselves and specialist institutions and persons or their family. Taking everything into account, the media should implement new methods for AIDS education in order to improve public knowledge of HIV/AIDS. Such findings show that media prevention campaigns should be encouraged and these have the potential role to limit the emergence of Turkey's HIV/AIDS epidemic. However, studies in Southeast Asia have shown that much of the media have done little to change existing cultural values and prejudice about the sexuality and situation of people who are living with HIV or AIDS [29]. In this study, overall, there were a lot of incorrect information, negative attitudes and misconceptions about HIV/AIDS. This is consistent with the findings of Agrawal et al (1999) [23], and can be attributed to the many false claims published in the media and other modes of advertisement.

In the present study, the respondents desired to learn more about HIV/AIDS (86.6%). This indicates that reports dealing with the rapid spread of AIDS in various populations, as well as in the Turkish population, have increased the level of anxiety over contagion among the respondents.

Sixty two percent of the sample believed they were at no risk of contracting HIV. This figure is comparable to the 2003 National HIV/AIDS and Reproductive Health Survey that reported 72% of the civilian population [30] and also Nwokoji and Ajuwon (2004)'s study [17]. This low perception of risk is probably influenced by the widespread denial of the existence of HIV in the country by governments or health authorities.

## Conclusion

The level of HIV/AIDS related knowledge was relatively high throughout our study, with most people showing positive attitudes. Misconceptions about AIDS still exist and need to be addressed by health education programs targeting those at higher risk. It has previously been shown that health education and sustained prevention efforts, such as the introduction of HIV/AIDS education into schools, mass-media campaigns promoting the use of condoms, and the wearing down of individuals' ability to deny risk towards patients with HIV or AIDS positive can be effective in changing people negative attitudes or misconceptions towards AIDS [31-33].

Being in total agreement with the opinion reported by Tebourski and Ben Alaya (2004) in their study [15], we would also like to state that success in increasing the publics' positive attitude to people with HIV/AIDS is vital to obtaining our goal of having a more effective control in the spreading of the disease.

## Competing interests

The author(s) declare that he has no competing interests

## Pre-publication history

The pre-publication history for this paper can be accessed here:


